# How to present an informative summary of findings table for systematic reviews of interventions: A tutorial

**DOI:** 10.1002/cesm.12093

**Published:** 2024-06-19

**Authors:** Jennifer Hilgart, Clare Miles, Jo‐Ana Chase

**Affiliations:** ^1^ Cochrane Evidence Production and Methods Directorate London UK

## Abstract

This tutorial provides guidance on creating clear and informative summary of findings tables for systematic reviews of interventions.

## Graphical abstract

This tutorial provides guidance on creating clear and informative summary of findings tables for systematic reviews of interventions. We will explain what information is required in the different sections of the table. In the accompanying micro‐learning module, you can test your knowledge of the key components of a summary of findings table and practice calculating absolute risks.

Summary of findings table micro learning module

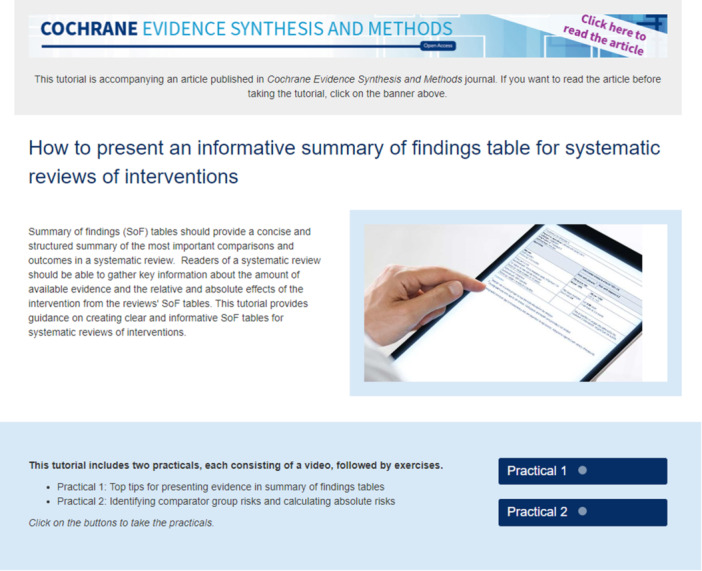

## 1 WHAT IS A SUMMARY OF FINDINGS (SOF) TABLE?

SoF tables present a useful format for providing readers with key information from a systematic review. They provide a concise and structured summary of the review's most important comparisons and outcomes. SoF tables can be included in many types of reviews, including diagnostic test accuracy reviews [1], qualitative evidence syntheses [2], and network meta‐analyses [3]. This tutorial provides guidance on creating clear and informative SoF tables for systematic reviews of interventions.

SoF tables also incorporate an assessment of the certainty of evidence using the GRADE (Grading of Recommendation, Assessment, Development, and Evaluation) approach. The certainty of the evidence (which can be assessed as high, moderate, low, or very low) is reported for each outcome in the SoF table. GRADE methods are beyond the scope of this article; however, there are many resources which provide guidance on GRADE (methods.cochrane.org/gradeing) and the GRADEPro GDT software (www.gradepro.org).

## 2 WHAT DO WE NEED TO CONSIDER WHEN CREATING SOF TABLES?

1. **Protocol considerations**
  Authors should begin prioritizing comparisons and outcomes for the SoF table at the protocol stage. The protocol should include a plan for at least one key SoF table(s) representing the most important comparison(s). Authors should also choose up to seven critical and important outcomes which will be summarized in the SoF table. The comparisons and outcomes chosen should align with the review's objectives and they could be chosen based on their importance for clinical decision making. For example, it may be that the intervention under review compared with usual care represents the most important choice for decision makers and patients. To ensure a balanced summary of the benefits and harms of an intervention, the table should include both desirable and undesirable outcomes. It is also important for authors to specify at which timepoints outcomes will be presented.
2. **Provide an informative PICO header and title**
  The title of the SoF table should clearly state the comparisons presented in the table and the population of interest. The scope of a systematic review (and each comparison within a review) can be described with a summary of the Population, Intervention, Comparison(s), and Outcomes, known as the PICO framework. The header of each SoF table should provide information about the PICO.
  Table 1 shows an example from a review of surgical versus nonsurgical interventions for displaced intra‐articular calcaneal fractures [4]. The main SoF table presented the comparison of surgical versus nonsurgical interventions. You can see here that the PICO header further clarifies that the population of interest is people aged over 14 years with displaced intra‐articular calcaneal fractures, the experimental intervention is surgical management, and the comparator intervention is nonsurgical management. The setting field of the header should state the setting addressed by the available evidence, which may be different to or narrower than that defined by the review question (e.g., intensive care in low‐middle income countries) so that readers can assess the applicability of the evidence to their own healthcare context. Where the studies were conducted in diverse places, broad indications of setting are acceptable (e.g., “in Europe and Asia”).
3. **Clearly define all outcomes including measures and time points**
  For this tutorial, we are concentrating on dichotomous and continuous outcome data, but other measures, such as time‐to‐event data [5] can also be reported in SoF tables. The first column of a SoF table provides information about the outcome. Authors should ensure that outcomes are fully defined including how the outcome was assessed and the time point (or time frame) at which the outcome was measured. Additionally, for continuous outcomes, authors should report the scales used to measure the outcome and the range of possible scores. Providing readers with this information will aid the interpretation of the effect estimates. For example, a mean difference (MD) of 2.5 points on a quality‐of‐life scale of 0–10 might represent an important difference, whereas the same difference on a scale of 0–100 may not. Table 2 demonstrates how outcomes can be clearly described in a SoF table [6]. For the continuous outcome of pain, the range of scores (0–100) and the direction of effect (lower = better) is reported. The minimal clinically important difference (MCID) is also provided which provides readers with information about whether the calculated MD is clinically important. For the dichotomous outcome of clinical improvement, information is provided about how the outcome was assessed. For both outcomes, the follow‐up time is specified.
4. **Provide comparator group risks**
  Comparator group risks (also known as assumed comparator risks or baseline risk) should illustrate what would typically happen when the comparator (control) intervention is used. Authors should ensure that the comparator group risk is completed and should report the source of this information in the SoF table footnotes. For dichotomous outcomes, the comparator group risk can be presented in the form of the number of people experiencing the event per 100 or 1000 people, depending on how frequent the event is. Authors can also use the median or mean control group risk as calculated from the studies included in the meta‐analysis. A comparator group risk for each study can be calculated (by dividing the number of events by the number of participants in the control group), then the mean or median risk can be identified. In situations where the baseline risk varies there may be a clinical rationale for presenting more than one risk. For example, a high and low‐risk value might be used where there are clinical reasons to consider high and low‐risk groups separately. The comparator risks could be selected from individual studies in the review which illustrate these risks, if representative studies are available. For example, a high risk value may be selected from a study that includes an older population and a low‐risk value might be selected from a study that includes a younger population. Authors could also identify other plausible estimates of risk, such as from high‐quality observational studies.
  When the included primary studies in a review have all used the same measure of a continuous outcome, the MD can be used as the effect estimate. The assumed risk in the control group can be reported as the mean, median, or range of values of the outcome from the included studies. Outcomes presented as SMDs may present further challenges of interpretability. Guidance on interpreting SMDs is available [7, 8, 9].
5. **Provide accurate absolute intervention group risks**
  Alongside measures of relative effects (such as risk ratios and odds ratios), SoF tables provide measures of absolute effects to facilitate interpretation of the review findings. Under the column/section labeled “absolute effects,” the comparator risk can be defined as the risk without the intervention and the corresponding risk can be defined as the risk with the intervention (or the impact of the intervention on risk). Authors should ensure that these numbers are accurate and informative. The direction of effect must also be consistent between the absolute risks and the relative risks. For the outcome of chronic pain shown in Table 1, the point estimates in the absolute risk and relative risk columns both show a reduced risk of pain with the surgical intervention. The 95% confidence intervals are also consistent with a reduced risk of pain with the intervention.
  Examples of control group risks and details of how to calculate intervention group risks are provided in the micro‐learning module which accompanies this tutorial.
6. **Write concise narrative summaries of evidence**
  Review authors may often encounter outcomes where a single pooled effect estimate from a meta‐analysis is not available for reporting in the summary of findings table. Outcomes may be reported incompletely, or clinical and methodological heterogeneity may mean a meta‐analysis is not appropriate. In these cases, the evidence is often summarized narratively or with other statistical synthesis methods [10]. The evidence should be reported in the SoF table as clear and concise narrative statements. Table 3 [11] demonstrates an example where authors narratively described findings for data they were unable to include in a meta‐analysis. If it is possible to include numerical data, this can also be reported as shown in Table 4 [12]. Additionally, authors should assess the certainty of the evidence using the GRADE approach [13].
7. **Provide additional information in the comments column**
  Authors may add text in the Comments column of the SoF table to facilitate interpretation of the data. For example, authors could include complementary estimates of intervention effects (e.g., number needed to treat for benefit and harm, risk difference expressed as percentages, continuous outcomes expressed in minimal important difference units) (see Table 2, outcome “Clinical Improvement”) or important caveats for the results (see Table 2, outcome “Pain”). If additional clarification is not beneficial, the Comments column may be left blank.

**Table 1:** Adapted extract from Lewis et al. [4].

**Surgical versus nonsurgical management for displaced intra‐articular calcaneal fractures**					
**Population:** People aged over 14 years with displaced intra‐articular calcaneal fractures					
**Setting:** Treated in hospital					
**Intervention:** Surgical management (ORIF, or closed reduction and percutaneous fixation)					
**Comparison:** Nonsurgical management (rest, elevation, ice; below‐knee plaster casts, and removable splints)					
**Outcomes**	**Anticipated absolute effects** ^a^ **(95% CI)**		**Relative effect (95% CI)**	**Number of participants (studies)**	**Comments**
	**Risk with non‐surgical treatment**	**Risk with surgery**			
Function in the short term	No studies reported function in the first 3 months after injury				
**Function in the long term** Measured using AOFAS score (0–100, higher scores indicate improved function) Follow‐up: 6–24 months	Mean scores in the nonsurgical groups ranged from 55 to 76.8.	**MD 6.58 higher** (1.04 higher to 12.12 higher)	‐	319 (5 studies)	The MCID for AOFAS in this fracture type is not established. MCIDs for other foot conditions range from 2.0 to 7.9.
**Chronic pain** Measured as the number of people experiencing pain Follow‐up: 6–24 months	670 per 1000^b^	375 per 1000 (248–563)	RR 0.56 (0.37–0.84)	175 (4 studies)	295 fewer people (95% CI 107–422 fewer) had chronic pain up to 24 months after surgery.

a The risk in the intervention group (and its 95% confidence interval [CI]) is based on the assumed risk in the comparison group and the relative effect of the intervention (and its 95% CI).

^b^ Derived from the pooled estimate of the nonsurgical treatment group.

**Table 2:** Adapted extract from Wieland et al. [6].

**Yoga compared with nonexercise control for chronic nonspecific low back pain**					
**Population:** People with chronic nonspecific low back pain					
**Setting:** Mix of participants seeking medical care and participants in the community					
**Intervention:** Yoga					
**Comparison:** Nonexercise (a waiting list, a minimal intervention [e.g., education], or usual care)					
**Outcomes**	**Anticipated absolute effects** ^a^ **(95% CI)**		**Relative effect (95% CI)**	**Number of participants (studies)**	**Comments**
	**Risk with nonexercise** ^b^	**Risk with yoga**			
**Pain** Assessed with numerical scale 0–100, lower = better, MCID 15 points Follow‐up: 3 months	Mean pain was 25.24 points (SD 12.23)	The MD was **4.53 points lower** in the yoga group (6.61 lower to 2.46 lower)	‐	946 (9 RCTs)	Yoga probably results in a slight reduction in pain but the difference in pain reduction between groups did not reach the predetermined clinically relevant difference (a 15‐point reduction on a 0–100 scale).
**Clinical improvement** Assessed as participant rating that back pain was improved or resolved. Follow‐up: 3 months	195 per 1000	454 per 1000 (284–723)	**RR 2.33** (1.46–3.71)	353 (4 RCTs)	Yoga may increase the risk of clinical improvement. Absolute difference 26% higher (9% higher to 53% higher); NNTB 4 (95% CI 2–12).

Abbreviations: RCTs, randomized controlled trials; RR, risk ratio.

a **The risk in the intervention group** (and its 95% confidence interval [CI]) is based on the assumed risk in the comparison group and the **relative effect** of the intervention (and its 95% CI).

^b^ When there was more than one study for an outcome, we chose the control group mean from the included study that had the most representative population and the greatest weight in the meta‐analysis. For pain this was Tilbrook 2011.

**Table 3:** Adapted extract from Southern et al. [11].

**High‐dose azithromycin versus low‐dose azithromycin for cystic fibrosis**					
**Population:** Children with cystic fibrosis					
**Setting:** Outpatient					
**Intervention:** High‐dose azithromycin (15 mg/kg/day)					
**Comparison:** Low‐dose azithromycin (5 mg/kg/day)					
**Outcomes**	**Anticipated absolute effects** ^a^ **(95% CI)**		**Relative effect (95% CI)**	**Number of participants (studies)**	**Comments**
	**Risk with low‐dose azithromycin**	**Risk with high‐dose azithromycin**			
FEV1% predicted: Follow‐up: 6 months	There was no difference in FEV1% predicted between baseline and 6 months in either group.		‐	47 (1 RCTs)	Results were given as change from baseline within groups and we could not enter the data into our analyses.

Abbreviations: FEV1%, forced expiratory volume in the first second; RCTs, randomized controlled trials.

a The risk in the intervention group (and its 95% confidence interval [CI]) is based on the assumed risk in the comparison group and the relative effect of the intervention (and its 95% CI).

**Table 4:** Adapted extract from Wagner et al. [12].

**Corticosteroids plus standard care compared to standard care (plus/minus placebo) for hospitalized and unvaccinated individuals with a confirmed or suspected diagnosis of symptomatic COVID‐19**					
**Population:** Hospitalized and unvaccinated individuals with a confirmed or suspected diagnosis of symptomatic COVID‐19					
**Setting:** Inpatient, ICU					
**Intervention:** Corticosteroids plus standard care					
**Comparison:** Standard care (plus/minus placebo)					
**Outcomes**	**Anticipated absolute effects** ^a^ **(95% CI)**		**Relative effect (95% CI)**	**Number of participants (studies)**	**Comments**
	**Risk with standard care**	**Risk with corticosteroids plus standard care**			
Adverse events (any grade) (follow‐up: during treatment)	We did not perform meta‐analyses because of high risk of bias, heterogeneous definitions, and underreporting. We only present descriptive statistics: Edalatifard 2020: RR 0.82 (95% CI 0.12–5.48); Tang 2021: RR 0.63 (95% CI 0.22–1.76); Tomazini 2020: RR 0.99 (95% CI 0.89–1.10).		‐	447 (3 RCTs)	The evidence is very uncertain about the effect of systemic corticosteroids on adverse events.
Hospital‐acquired infections (follow up: during treatment)	We did not perform meta‐analyses because of high risk of bias, heterogeneous definitions, and underreporting. We present descriptive statistics only: Corral‐Gudino 2021: RR 4.14 (95% CI 0.51–33.49); Dequin 2020: RR 0.90 (95% CI 0.60–1.34); Tang 2021: RR 2.00 (95% CI 0.19–21.24); Tomazini 2020: RR 0.75 (95% CI 0.50–1.15).		‐	598 (4 RCTs)	The evidence is very uncertain about the effect of systemic corticosteroids on hospital‐acquired infections.

Abbreviation: RCTs, randomized controlled trials.

a **The risk in the intervention group** (and its 95% confidence interval [CI]) is based on the assumed risk in the comparison group and the **relative effect** of the intervention (and its 95% CI).

## 3 SUMMARY

Systematic reviews of interventions can support informed healthcare practices and decision‐making. SoF tables are useful tools for distilling key information from these reviews into a succinct and accessible format. This tutorial has outlined guidance for presenting accurate and informative SoF tables.

## AUTHOR CONTRIBUTIONS

**Jennifer Hilgart**: Conceptualization; methodology; project administration; supervision; writing—original draft; writing—review & editing. **Clare Miles**: Conceptualization; supervision; writing—review & editing. **Jo‐Ana Chase**: Conceptualization; supervision; writing—review & editing.

## CONFLICT OF INTEREST STATEMENT

All authors are employed by Cochrane.

## PEER REVIEW

The peer review history for this article is available at https://www.webofscience.com/api/gateway/wos/peer-review/10.1002/cesm.12093.

## FURTHER READING AND ONLINE CONTENT

More information on completing summary of findings tables and grading the certainty of the evidence can be found in Chapter 14 of The Cochrane Handbook for Systematic Reviews of Interventions [14].

Cochrane Training has produced a micro‐learning module on creating informative summary of findings tables to accompany this article (https://links.cochrane.org/cesm/tutorials/sof-table-for-interventions). Additional references for content in the micro‐learning module [15, 16, 17, 18, 19, 20, 21].

## Data Availability

Data sharing is not applicable to this article as no new data were created or analyzed for this tutorial. References have been provided for the published Cochrane reviews which have been used as examples in this tutorial. Example reviews may have been adapted slightly for training purposes.
